# Common genetic variants and pathways in diabetes and associated complications and vulnerability of populations with different ethnic origins

**DOI:** 10.1038/s41598-021-86801-2

**Published:** 2021-04-05

**Authors:** Sabrina Samad Shoily, Tamim Ahsan, Kaniz Fatema, Abu Ashfaqur Sajib

**Affiliations:** 1 Department of Genetic Engineering and Biotechnology, University of Dhaka, Dhaka, Bangladesh; 2 Department of Genetic Engineering & Biotechnology, Bangabandhu Sheikh Mujibur Rahman Maritime University, Dhaka, Bangladesh

**Keywords:** Computational biology and bioinformatics, Genetics, Biomarkers, Diseases, Endocrinology

## Abstract

Diabetes mellitus is a complex and heterogeneous metabolic disorder which is often pre- or post-existent with complications such as cardiovascular disease, hypertension, inflammation, chronic kidney disease, diabetic retino- and nephropathies. However, the frequencies of these co-morbidities vary among individuals and across populations. It is, therefore, not unlikely that certain genetic variants might commonly contribute to these conditions. Here, we identified four single nucleotide polymorphisms (rs5186, rs1800795, rs1799983 and rs1800629 in AGTR1, IL6, NOS3 and TNFA genes, respectively) to be commonly associated with each of these conditions. We explored their possible interplay in diabetes and associated complications. The variant allele and haplotype frequencies at these polymorphic loci vary among different super-populations (African, European, admixed Americans, South and East Asians). The variant alleles are particularly highly prevalent in different European and admixed American populations. Differential distribution of these variants in different ethnic groups suggests that certain drugs might be more effective in selective populations rather than all. Therefore, population specific genetic architectures should be considered before considering a drug for these conditions.

## Introduction

Diabetes is a metabolic disorder characterized primarily by chronic hyperglycemia, which results from the inability of pancreas to produce and/or secrete enough insulin and/or resistance to insulin in the peripheral tissues^1^. Insulin is a master regulator of cellular metabolism and its reduced supply and/or action has pleiotropic effects on metabolic pathways^1,2^. According to International Diabetes Federation (IDF) Atlas (9^th^ edition), in 2019 approximately 463 million adults between 20 and 79 years of age had diabetes and it caused 4.2 million deaths^3^. The number of individuals with this debilitating disease is expected to reach 700 million in 2045^3^.

The leading cause of morbidity and mortality among diabetic individuals is long-term micro- and macro-vascular complications^4^. Microangiopathy is a major characteristic of diabetes, which is seen more prominently in the eyes (diabetic retinopathy) and in the kidneys (diabetic nephropathy)^5^. Even in the early stages of diabetes, microvascular damages might be demonstrable in the form of basement membrane thickening of small blood vessel^5^. The risk of vascular complications in diabetic individuals increases with aggregated comorbidities^4^. Hypertension or sustained high blood pressure (≥ 140/90 mmHg) is strongly associated with diabetic complications and known as a risk factor for atherosclerotic cardiovascular disease (ASCVD) and microvascular complications^6^. In people with diabetes, anti-hypertensive medications reduce microvascular complications as well as ASCVD events^7^. Hyperglycemia and hypertension are two prominent risk factors for chronic kidney disease (CKD), which is developed in approximately 40% of the diabetic patients^8^. In both developed and developing countries, the leading causes of end-stage renal disease (ESRD) are diabetic nephropathy (DN) and hypertensive nephropathy^9^. An important characteristic of diabetic kidney disease is albuminuria, which is accompanied with increased blood pressure in chronic kidney disease progression^10^. Hypertension not only contributes to CKD progression, but also causes damage to the kidney by arterial stiffness, endothelial dysfunction, increased renin–angiotensin–aldosterone system (RAAS) and increased salt retention^11^. RAAS is also associated with pathophysiology of diabetes and its micro- and macrovascular complications^12^. Additionally, increased amount of urea in blood in individuals with chronic kidney disease causes defect in insulin secretion from the pancreatic β cells^13^. Such interplay among diabetes and associated complications hint towards common contributing factors.

Single nucleotide polymorphisms (SNPs) can contribute to development and progression of diseases by influencing expression of gene, stability of messenger RNA (mRNA) and/or translational efficiency^14^. For example, diabetes associated risk alleles have been reported in genes that regulate pancreatic beta cell development and function, insulin gene expression, secretion and action^15–18^. Many SNPs have also been associated with hypertension^19^, inflammation^20^, chronic kidney disease^21^, cardiovascular disease^22^, diabetic retino-^23^ and nephropathies^24^. Prevalence of diabetes associated complications varies among different ethnic groups^25–27^. Disparities in the variant allele frequencies among different ethnic regions may contribute to varied disease susceptibilities^28^. Early detection of diabetes and risk measurement is of significant importance to minimize these complications^3^.

In this study, the common SNPs that are associated with diabetes, diabetic nephro- and retinopathies, cardiovascular disease, inflammation, hypertension and kidney diseases were identified and population specific variant allele and haplotype frequencies at these loci were discerned. In addition, the genes that harbor these variants and associated pathways were investigated to understand their roles in the etiology of diabetes associated complications.

## Result

### Common genetic variants in diabetes and associated complications

Only four SNPs (rs5186, rs1800629, rs1799983, and rs1800795) were found to have association with diabetes, cardiovascular diseases, diabetic nephropathy, diabetic retinopathy, hypertension, inflammation, and kidney diseases. These SNPs reside in four different genes- Angiotensin II receptor type 1 (AGTR1), Tumor necrosis factor alpha (TNFA), Nitric oxide synthase 3 (NOS3, also known as endothelial nitric oxide synthase or eNOS) and Interleukin-6 (IL6)) on three different chromosomes (Table 1). rs1800629 and rs1800795 reside in the upstream region of TNFA and IL6, while rs5186 and rs1799983 cause variations in the 3′ prime untranslated region (UTR) and coding region (missense) of AGTR1 and NOS3 genes, respectively. rs1799983 leads to transversion from guanine (G) to thymine (T) at nucleotide position 894 (G894T) resulting in the replacement of glutamic acid by aspartic acid at codon 298 (Glu298Asp).

**Table 1:** Frequencies of rs5186, rs1800629, rs1799983 and rs1800795 variant alleles and haplotypes in world populations.

SNP ID		rs5186	rs1800629	rs1799983	rs1800795	Haplotype (rs1800795_rs1799983)			
Gene		AGTR1	TNFA	NOS3	IL-6				
Chr:position		chr3: 148459988	chr7: 150696111	chr7: 150696111	chr7: 22766645				
Major allele		A	G	G	G				
Minor allele		C	A	T	C				
Functional consequence		3′ prime UTR variant	Upstream transcript variant	Missense variant	Upstream transcript variant				
Super-population	Sub-population	Minor (variant) allele frequency							
All populations (ALL)*	All populations (ALL)	0.118	0.090	0.176	0.141	G_G	G_T	C_G	C_T
African (AFR)	African (AFR)	0.020	0.120	0.070	0.018	0.914	0.068	0.016	0.002
	Yoruba in Ibadan, Nigeria (YRI)	0.014	0.102	0.056	0.000	0.944	0.056		
	Luhya in Webuye, Kenya (LWK)	0.005	0.086	0.035	0.000	0.965	0.035		
	Gambian in Western Divisions in the Gambia (GWD)	0.013	0.142	0.075	0.004	0.920	0.075	0.004	
	Mende in Sierra Leone (MSL)	0.012	0.159	0.047	0.000	0.953	0.047		
	Esan in Nigeria (ESN)	0.005	0.126	0.101	0.000	0.899	0.101		
	Americans of African Ancestry in SW USA (ASW)	0.090	0.074	0.107	0.090	0.803	0.107	0.090	
	African Caribbeans in Barbados (ACB)	0.031	0.135	0.083	0.063	0.870	0.068	0.047	0.016
Admixed American (AMR)	Admixed American (AMR)	***0.233***	0.069	***0.215***	0.184	0.654	0.161	0.131	0.053
	Mexican Ancestry from Los Angeles USA (MXL)	***0.266***	0.055	0.195	0.133	0.703	0.164	0.102	0.031
	Puerto Ricans from Puerto Rico (PUR)	***0.212***	0.087	***0.284***	***0.231***	0.572	0.197	0.144	0.087
	Colombians from Medellin, Colombia (CLM)	***0.207***	0.069	***0.266***	***0.287***	0.527	0.207	0.186	0.080
	Peruvians from Lima, Peru (PEL)	***0.265***	0.059	0.088	0.053	0.859	0.088	0.053	
East Asian (EAS)	East Asian (EAS)	0.060	0.059	0.130	0.001	0.869	0.130	0.001	
	Han Chinese in Beijing, China (CHB)	0.053	0.092	0.136	0.000	0.864	0.136		
	Japanese in Tokyo, Japan (JPT)	0.063	0.019	0.082	0.000	0.918	0.082		
	Southern Han Chinese (CHS)	0.091	0.057	0.148	0.000	0.852	0.148		
	Chinese Dai in Xishuangbanna, China (CDX)	0.038	0.070	0.129	0.000	0.871	0.129		
	Kinh in Ho Chi Minh City, Vietnam (KHV)	0.051	0.056	0.157	0.005	0.838	0.157	0.005	
European (EUR)	European (EUR)	***0.272***	0.134	***0.344***	***0.416***	0.391	0.265	0.194	***0.150***
	Utah Residents (CEPH) with Northern and Western European Ancestry (CEU)	***0.298***	0.187	***0.364***	***0.485***	0.333	0.303	0.182	***0.182***
	Toscani in Italia (TSI)	***0.238***	0.094	***0.397***	***0.355***	0.402	0.243	0.201	***0.154***
	Finnish in Finland (FIN)	***0.207***	0.126	***0.232***	***0.455***	0.414	0.354	0.131	***0.101***
	British in England and Scotland (GBR)	***0.313***	0.121	***0.335***	***0.412***	0.385	0.280	0.203	***0.132***
	Iberian Population in Spain (IBS)	***0.308***	0.145	***0.383***	***0.351***	0.444	0.206	0.178	***0.173***
South Asian (SAS)	South Asian (SAS)	0.069	0.053	0.168	0.139	0.714	0.147	0.119	0.020
	Gujarati Indian from Houston, Texas (GIH)	0.092	0.049	0.146	0.155	0.723	0.131	0.121	0.024
	Punjabi from Lahore, Pakistan (PJL)	0.089	0.057	0.151	0.130	0.745	0.125	0.104	0.026
	Bengali from Bangladesh (BEB)	0.052	0.023	0.157	0.116	0.756	0.128	0.087	0.029
	Sri Lankan Tamil from the UK (STU)	0.059	0.093	0.177	0.113	0.721	0.167	0.103	0.010
	Indian Telugu from the UK (ITU)	0.049	0.039	***0.206***	0.177	0.632	0.191	0.162	0.015

### Distribution of the common genetic variants in different populations

Minor (variant) allele frequencies (MAFs) of these SNPs in world populations are shown in Table 1. SNPs with MAFs ≥ 0.2 are shown as bold italic, while MAFs with values ≥ 0.3 are underlined in Table 1. Frequencies of these variants follow similar pattern among the constituent populations within a super-population, but vary among the super-populations. For example, compared to other populations, MAFs of all the variants are relatively low in the African populations with no value ≥ 0.2. In four African populations (Yoruba in Ibadan, Nigeria (YRI), Luhya in Webuye, Kenya (LWK), Mende in Sierra Leone (MSL), Esan in Nigeria (ESN)) the variant allele at rs1800795 is non-existent (MAF = 0.00). The MAFs at these loci in the East Asian populations are also very low. At the rs1800795 locus, the MAFs range from 0.00 to 0.005 in these populations. Frequency of the C_T haplotype, which is formed by two variant alleles at rs1800795 and rs1799983 loci, in the African and the East Asian populations is zero. In the South Asian populations, the variant allele frequencies at these loci are not as low as the African and the East Asian populations, but all these MAFs, except one, are smaller than 0.2.

In the admixed American (AMR) populations, the MAFs at these loci are much higher than the African, East- and South Asian populations. The MAFs are ≥ 0.2 at rs5186 in the four admixed American (AMR) populations. Puerto Rican (PUR) and Colombian (CLM) populations within AMR super-population have MAF > 0.2 at rs1800795 as well. The overall variant allele frequency of rs1799983 in AMR super-population is > 0.2, although the MAFs are greater than 0.2 in only two constituent populations (PUR and CLM).

The European sub-populations have high MAF values for rs5186, rs1799983 and rs1800795. The overall MAFs at rs5186, rs1799983 and rs1800795 in the European super-population are 0.272, 0.344 and 0.416, respectively. Particularly at the rs1800795 locus, the MAFs are much higher in the European populations (ranging from 0.351 to 0.485). Both IL6 (rs1800795) and NOS3 (rs1799983) reside on chromosome 7. The haplotype harboring the variant alleles (C_T) at both rs1800795 and rs1799983 loci are present at frequencies > 0.1 in the European populations only.

### Interaction among the candidate genes

**Figure 1:**
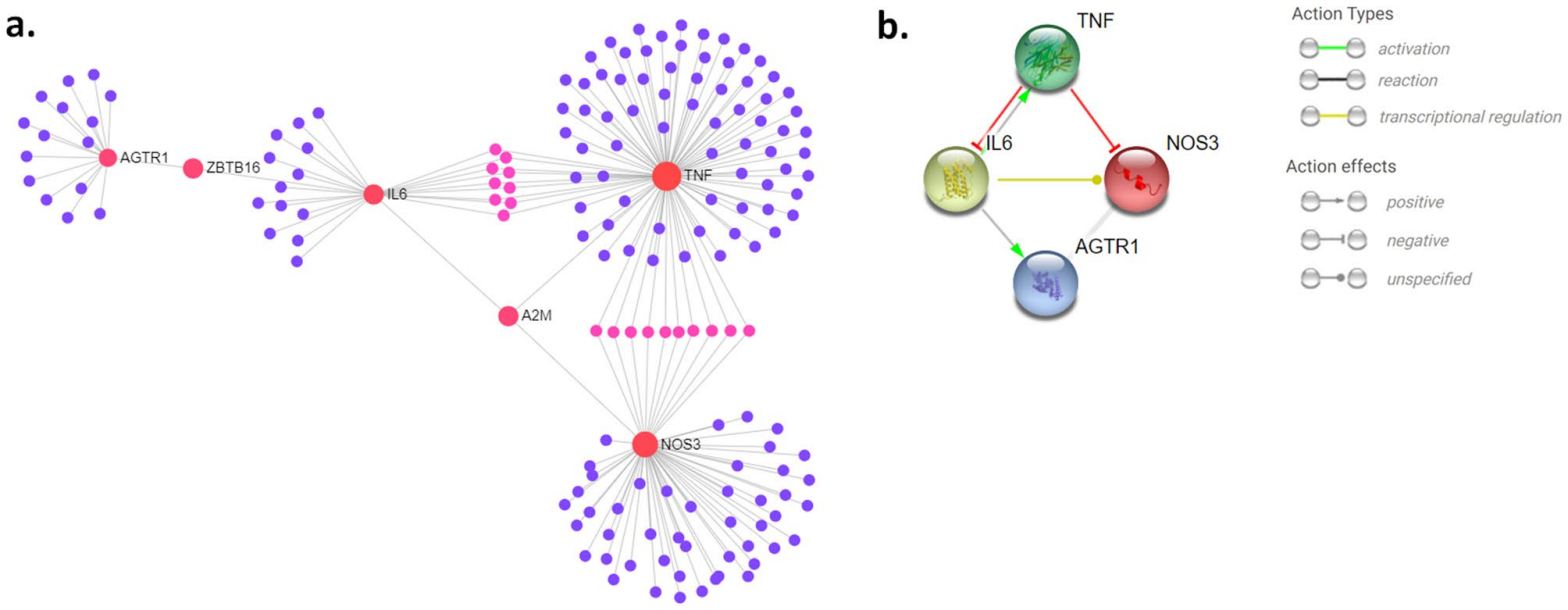
Protein–protein interaction networks. **(A)** Network derived from IMEx interactome database (using NetworkAnalyst^120^ web-based visual analytics platform) shows interactions among AGTR1, IL6, NOS3 and TNFA. **(B)** Network derived through STRING database^30^ shows the nature of interactions among AGTR1, IL6, NOS3 and TNFA.

The protein–protein interaction (PPI) networks generated through IMEx^29^ and STRING^30^ indicate interactions among these gene encoded proteins (Fig. 1). However, the nature of interactions (direct and indirect) depicted by IMEx and STRING are somewhat different. These differences might have derived from the nature of these databases. IMEx consortium annotates experimental interaction evidences directly from the source publications and provide curated non-redundant set of physical and molecular interaction data^29^. The STRING database, on the other hand, collects and integrates all publicly available protein–protein interaction information as well as predicted interactions along with annotated pathway knowledge and text-mining results to provide direct (physical) as well as indirect (functional) interactions^30^.

Based on the PPI network predicted using STRING, TNFA inhibits NOS3 and IL6. It has no known direct functional effect on AGTR1, which is also seen in the network derived with IMEx. IL6 activates TNFA and AGTR1 and regulates transcription of NOS3. In the IMEx-derived network, zinc finger and BTB domain-containing protein 16 (ZBTB16), a transcription factor with nine zinc fingers, connects AGTR1 and IL6, whereas alpha-2-macroglobulin (A2M) connects IL6, TNFA and NOS3.

Pathways that incorporate these proteins (AGTR1, IL6, NOS3 and TNFA) are listed in Table 2. These pathways were identified from the KEGG database based on the PPI networks derived through IMEx and SRTING. Among these IL6, NOS3, TNFA and AGTR1 plays role in 9, 8, 7, and 5 pathways, respectively. Only the AGE-RAGE signaling pathway in diabetic complications accommodates all four candidate genes.

**Table 2:** Pathways predicted to be affected by rs5186, rs1799983, rs1800795 and rs1800629 variants.

Pathway	p-value	False discovery rate (FDR)	Participating proteins
AGE-RAGE signaling pathway in diabetic complications	2.63E−08	8.36E−06	AGTR1, IL-6, NOS3, TNF
Insulin resistance	1.05E−05	0.00167	IL-6, NOS3, TNF
HIF-1 signaling pathway	0.000976	0.0197	IL-6, NOS3
IL-17 signaling pathway	0.000844	0.0197	IL-6, TNF
Toll-like receptor signaling pathway	0.00106	0.0197	IL-6, TNF
TNF signaling pathway	0.00118	0.0208	IL-6, TNF
Sphingolipid signaling pathway	0.00138	0.0231	NOS3, TNF
Apelin signaling pathway	0.00182	0.0284	AGTR1, NOS3
cGMP-PKG signaling pathway	0.00267	0.0344	AGTR1, NOS3
NOD-like receptor signaling pathway	0.00306	0.0365	IL-6, TNF
Calcium signaling pathway	0.00341	0.0388	AGTR1, NOS3

### Distribution of the other potentially deleterious genetic variants of AGTR1, IL6, NOS3 and TNFA in world populations

We used the Ensembl Allele Frequency Calculator, SIFT and PolyPhen-2 tools to retrieve frequencies of the other potentially deleterious variants in AGTR1, IL6, NOS3 and TNFA genes in worldwide populations based on the data in the 1000 Genomes Project database (Supplementary Table S1). Except a few, majority of these potentially deleterious variants are either absent or have very low frequencies in most populations. Among these variants, rs2069842 and rs2069849 are present at relatively high frequencies particularly in the African populations.

### Drug-responsiveness of rs5186, rs1800629, rs1799983 and rs1800795 variants

As AGRT1, IL6, NOS3 and TNFA genes are common candidates for diabetes and associated complications, drugs that target these might mitigate multiple risk factors simultaneously. Table 3 provides the list of FDA approved drugs which target these gene products. Interactions of the SNPs with these drugs are shown in Table 4. Interestingly, rs1045642, a variant in the multi-drug transporter ABCB1 (also known as MDR1 and P-gp), is in strong linkage disequilibrium with rs1800795 (IL6) and rs1799983 (NOS3) (Supplementary Table S2). rs1045642 is associated with hypertension and modulates responses to anti-hypertensive drugs^31,32^. Haplotypes with ≥ 2 variant alleles at rs1800795, rs1045642 andrs1799983 have high frequencies in the European populations (Supplementary Table S2).

**Table 3:** List of approved drugs that target AGRT1, IL-6, NOS3 and TNFA.

Gene	Action	Drug (source: DrugBank and DGIdb)
AGTR1	Antagonist	Valsartan, Olmesartan, Losartan, Candesartan cilexetil, Eprosartan, Telmisartan, Irbesartan, Azilsartan medoxomil
	Agonist	Angiotensin II
NOS3	Inhibitor	Miconazole
	Inducer	Levamlodipine, Pentaerythritol tetranitrate
	Agonist	Levamlodipine
IL-6	Inhibitor/antibody	Tocilizumab
	Antagonist/antibody	Sarilumab
TNFA	Antagonist	Glycyrrhizic acid
	Inhibitor/antibody	Golimumab, Adalimumab, Etanercept, Polaprezinc, Pseudoephedrine, Pomalidomide, Amrinone, Chloroquine, Thalidomide, Infliximab
	Inducer	Bryostatin 1
	Neutralizer	Certolizumab pegol

**Table 4:** Variant alleles of AGTR1, IL6, NOS3 and TNFA that are known to modulate responses to approved drugs.

SNP_ID	Gene	Chromosome	Drug	PharmGKB clinical annotation	Type	Major allele	Minor allele	Minor (variant) allele frequencies					
								ALL	AFR	AMR	EAS	EUR	SAS
**rs1800795**	**IL6**	7:22,727,026	Adalimumab, etanercept, Infliximab	Level 3	Efficacy	G	C	0.141	0.018	0.184	0.001	***0.416***	0.139
**rs1800629**	**TNF**	6:31,575,254	Etanercept	Level 2B	Efficacy	G	A	0.090	0.120	0.069	0.059	0.134	0.053
rs1799724	**TNF**	6:31,574,705	Infliximab	Level 3	Efficacy	C	T	0.099	0.024	0.183	0.125	0.094	0.119
rs361525	**TNF**	6:31,575,324	Infliximab	Level 3	Efficacy	G	A	0.061	0.038	0.082	0.031	0.064	0.105
rs12721226	**AGTR1**	3:148,741,522	Losartan	Level 4	Efficacy	G	A	0.001	0.000	0.000	0.002	0.000	0.003
rs1045642	ABCB1	7:87,509,329	Losartan	Level 3	Efficacy	G	A	***0.395***	0.150	***0.428***	***0.398***	***0.518***	***0.575***
**rs5186**	**AGTR1**	3:148,742,201	Losartan, Angiotensin II	Level 3	Efficacy	A	C	0.118	0.020	***0.233***	0.060	***0.272***	0.069

SNPs and genes that are associated with diabetes and its complications are written in bold letters. MAFs ≥ 0.2 are shown in bold italic font.

## Discussion

### Relation of rs5186, rs1800629, rs1799983 and rs1800795 variants to diabetes and associated complications

Diabetes is a multifactorial endocrine disorder which heightens the chances of developing other complex conditions^33^. It is, therefore, not impossible to have an underlying link among these conditions. Determining these underlying common genetic factors may pave ways for devising more effective diagnostic and prognostic strategies, improve treatment regimes and increase individualized drug efficacies. Interethnic differences in the frequencies of such common disease causing genetic factors may be accounted for variation in the prevalence of disease conditions in different regions. Here, we identified four SNPs (rs5186, rs1800629, rs1799983, rs1800795) that are associated with diabetes, cardiovascular disease, diabetic retino- and nephropathies, hypertension, inflammation and kidney diseases.

rs5186 is located at position 1166 in the 3′ untranslated region of AGTR1, which encodes the angiotensin II receptor type 1^34^. Frequencies of the variant allele (C) at rs5186 are high in the European and the admixed American populations (Table 1). Complementary base pairing of microRNA-155 (miR-155) suppresses translation of AGTR1 mRNA when the wild type allele (A) is present at the rs5186 locus^34^. The C allele prevents this base pairing, which leads to increased AGTR1 protein levels^34^. In the South Asian population, A allele of rs5186 acts as a protective factor against renal disease development^34^. Compared to the AA genotype at this locus, the CC genotype is related to diabetic nephropathy^35,36^. Elevated AGTR1 protein lead to increased activation of renin–angiotensin–aldosterone system (RAAS) as angiotensin II binds primarily to AGTR1 to exert its actions^37^. Pancreatic RAAS plays an important role in the pathophysiology of diabetes^38^. Activation of AGTR1 can cause pancreatic β-cell death via NADPH oxidase induction and ROS generation^39^. Increased activation of RAAS is also associated with hypertension^40^, regulation of inflammatory cascade^41^, progression to chronic kidney disease (CKD) and coronary heart disease (CHD)^42^. The exact mechanism of RAAS in the development of diabetic retinopathy is still not fully elucidated, but its regulatory role in vascular hydrodynamics and upregulation of its components in diabetic retinopathy point towards its involvement in the onset and progression of this disease^43^. High prevalence of rs5186 variant allele may result in higher incidences of the above-mentioned complications in the European and admixed American populations through over-activation of RAAS.

TNFA is a pro-inflammatory cytokine produced by a variety of cell-types such as lymphocytes, macrophages and adipocytes^44^. This immunoregulatory cytokine stimulates many other cytokines leading to a cytokine cascade, which causes inflammation^45^. TNFA rs1800629 (-308G > A) variant is located upstream of the coding region of the gene and known to influence TNFA levels^45^. The -308A polymorphism affects transcription factor binding, which can lead to TNF gene transcription causing inappropriate and excessive TNFA production^46^. Higher levels of this cytokine have been found in diabetic patients compared to non-diabetic controls^44^. Moreover, serum level of TNFA is significantly higher in obese diabetic patients^47^. It plays an important pathophysiological role in the development of insulin resistance, especially in people with high Body Mass Index (BMI)^48^. Overweight and obesity are highly prevalent in Europe^49^. Interestingly, among the five super-populations in this study, European super-population has the largest MAF at rs1800629 locus (Table 1). TNFA also plays a key regulatory role in endothelial dysfunction in diabetes^50^. Local activation of this cytokine within fibrovascular membranes of eyes plays an important role in the development of the proliferation phase of diabetic retinopathy^51^. Its serum level is more elevated in diabetic nephropathy patients compared to diabetic patients^52^.

Among the five super-populations, the largest MAF at rs1800795 is present in the European populations. rs1800795 (-174 G > C) is a variant in the promoter region of IL6 gene, which is a classic inflammatory cytokine^53^. rs1800795 modulates transcription rate of IL6^53^. Carriers of rs1800795 C allele have higher plasma level of IL6^54^. This variant allele is more common in diabetic patients compared to healthy controls^55^. In addition, this allele increases the risk of obesity-related metabolic disorders, especially insulin resistance, in people with excessive body-weight^56^. High prevalence of overweight and obesity in the European populations^49^ may exacerbate the effect of this variant allele, which is highly prevalent at this locus. rs1870095 C allele can also lead to a greater predisposition to atherosclerosis and myocardial or vascular injury^54^. Intraocular accumulation of IL6 is observed in proliferative diabetic retinopathy, although its involvement might not occur until the later stage of this disease^57^. Renal expression of pro-inflammatory cytokines including both TNFA and IL6 are increased in diabetic nephropathy^58^.

The variant allele frequencies at the rs1799983 locus in NOS3 gene are high in the European and admixed American populations. This missense variant resides in the exon 7 of NOS3 gene. This gene is constitutively expressed to make of nitric oxide (NO) in the endothelial cells and plays role in vasodilation and blood pressure regulation^59^. The variant (T) allele at this position is associated with significantly reduced basal NO production^60^. Decreased NO level can lead to hypertension as it plays a role in maintaining normal blood pressure by dilating smooth muscles to allow relaxed blood flow^61^. Reduced level of NO is also associated with abnormal levels of blood lipids^60^. Additionally, without the antioxidative role of NO, the hydroxyl radicals increase, which contribute to endothelial dysfunction^60^. Dyslipidemia and endothelial dysfunction are associated with atherosclerosis^60^. The oxidative effects by low levels of NO might also play a role in insulin resistance and diabetes^62^. NOS knocked-out diabetic mice exhibit accelerated retinopathy^63^. Reduced level of NO has been reported in progressive diabetic nephropathy and chronic kidney disease (CKD)^64^.

The rs5186, rs1800629, rs1799983 and rs1800795 variants in the AGTR1, TNFA, NOS3 and IL6, respectively, are therefore relevant to diabetes and associated complications. These four genes are involved in AGE-RAGE signaling pathway (Table 2), which is known to cause diabetes associated complications^65,66^. Hyperglycemia accelerates the formation of advanced glycation end products (AGEs), which interact with their major cell surface signal transduction receptor for AGE (RAGE) on smooth muscle cells, endothelial cells and monocytes, thereby inducing a wide range of signaling pathways^65,67^. Ligand-engagement of RAGE triggers activation of NADPH oxidase, which leads to generation of reactive oxygen species (ROS), that in turn can activate renin-angiotensin system (RAS)^68^. Pathophysiological cross-talk between AGE-RAGE axis and RAS can contribute to the progression of diabetes-associated vascular damage^69^. RAS activation and subsequent angiotensin II production play an important role in hypertension as well as renal and cardiac fibrosis^70^. Additionally, angiotensin II stimulates the expression of transforming growth factor-β (TGF-β) in the kidney, which play significant roles in the development of diabetic nephropathy^71^.

ROS also triggers nuclear factor kappa-light-chain-enhancer of activated B cells (NF-κB), which is a key player in cytokine and inflammatory mediator expression that results in micro-vasculopathy^65^. The up-regulation of transcription factor NF-κB triggers the secretion of pro-inflammatory cytokines such as IL6 and TNFA^72^. ROS generated by AGE-RAGE interaction can activate protein kinase C (PKC), which upregulates TGF-β, VEGF, NF-κB, NADPH oxidase and down-regulates endothelial nitric oxide synthase (NOS3) causing reduced production of NO, and thus affect blood flow and capillary permeability^73^. Furthermore, interaction between TNFA and IL6 increases oxidative stress, reduce NOS3 phosphorylation and contribute to coronary endothelial dysfunction in diabetic mice^50^. Thus, up-regulation of AGTR1, IL6 and TNFA and reduced production of NO in the context of AGE-RAGE pathway contribute to diabetes and its complications. As mentioned earlier, the variant alleles at rs5186, rs1800629, rs1799983 and rs11800795 loci cause such up- and down-regulations in the expression of corresponding genes. Thereby, presence of these variant alleles may exacerbate diabetic complications.

IL6, TNFA and NOS3 are associated with insulin resistance (Table 2). Insulin resistance (IR) is characterized as a condition in which sensitivity to insulin and its downstream signaling pathways is decreased in three primary insulin sensitive metabolic tissues– skeletal muscle, liver, and white adipose under normal serum glucose concentrations^74^. By increasing STAT3 (signal transducer and activator of transcription 3) phosphorylation, IL6 enhances the expression of suppressor of cytokine signaling 3 (SOCS3), which directly interferes with insulin signaling by binding to insulin receptor (IR), preventing its interaction with insulin receptor substrate-1 (IRS-1) (primarily in skeletal muscle and adipose tissue) or IRS-2 (primarily in liver) and targeting IRS-1 and IRS-2 for degradation^75^. IL6 also contributes to insulin resistance by inducing TLR4 expression in skeletal muscle via STAT3^76^. In addition, IL6 may impair insulin mediated IRS-1/PI3-kinase/Akt pathway by activating c-Jun NH2-terminal kinase (JNK) and extracellular signal-regulated kinase-1/2 (ERK1/2), which in turn induce serine phosphorylation of IRS-1^77^. Serine phosphorylation of IRS-1 results in reduced insulin receptor kinase activity^78^. These disruptions in early events of insulin pathway ultimately leads to insulin resistance and attenuated GLUT-4 mediated glucose uptake^75^. TNFA impairs insulin signal transduction in skeletal muscle by causing increased serine phosphorylation and reduced tyrosine phosphorylation of IRS-1, which leads to interference with GLUT4 translocation due to impaired phosphorylation of Akt substrate of 160 kDa (AS160)^79^.

As depicted in the protein–protein interaction networks (Fig. 1B), TNFA inhibits NOS3 activity, whereas IL6 regulates of NOS3 and AGTR1. In cultured vascular smooth muscle cell, IL6 have been shown to enhance expression of AGTR1^80^. So, the complications arising from increased level of AGTR1 may be mediated by IL6. Based on the PPI network (Fig. 1), TNFA and IL6 can influence each-others activation. In obesity, production of IL6 is stimulated by both IL-1β and TNFA^81^. This inductive role of IL-1β is outlined in the supplementary Fig. S1. This proinflammatory cytokine along with elevated IL6 inhibits insulin signaling pathways and contributes to the development of diabetes^82^.

As shown in Table 2, IL6 and NOS3 are part of the Hypoxia-inducible factor 1 (HIF-1) signaling pathway. HIF-1 binds DNA at the hypoxia response element (HRE) and activates expression of several hypoxia induced genes involved in glucose metabolism, angiogenesis, cell proliferation, survival and metastasis^83^. HIF-1 binds to the HRE in the NOS3 gene promoter causing its increased expression which in turn elevates NO levels in the endothelial cells, and thus contributes to vasodilation and vascular permeability^84^. HIF-1α (one of the constituent subunits of HIF-1) expression is elevated by activated IL6/STAT3 pathway^85^. So, increased IL6 expression in presence of the rs1800795 variant (C) allele (as previously discussed) may stimulate HIF-1 expression, which transcriptionally activates several angiogenic genes (such as VEGF) and their receptors^86^. VEGF level is significantly elevated in diabetic patients with different micro- and macrovascular complications and it is a principle mediator of diabetic retinopathy^87^.

HIF-1α contributes to the hypoxia induced regulation of apelin and the apelin receptor^88^ to stimulate cell proliferation and angiogenesis via PI3K-Akt and mTOR mediated processes^89^. Both IMEx and STRING predicted PPI networks found AGTR1 and NOS3 to be associated with the Apelin signaling pathway, cGMP-PKG signaling pathway and calcium signaling pathway (Table 2). Apelin signaling pathway is involved in normal vascular development and in regulation of NO-dependent vasodilatation^88^. Reduced production of NO resulting from NOS3 rs1799983 T allele might interfere with this apelin regulated vasodilation.

TNFA and IL6, the two cytokines are involved in IL-17 signaling pathway, Toll-like receptor signaling pathway and TNF signaling pathway (Table 2). IL-17 stimulates production of proinflammatory cytokines (IL-1β, IL6 and TNFA), which contributes to the induction of insulin resistance and development of diabetes^90^. IL6 activated STAT3 increases TLR4 gene expression^76^. TLR4 activation can contribute to insulin resistance by proinflammatory cytokines in diabetes^91^. Thus, rs180079 variant mediated increased IL6 expression may contribute to insulin resistance via TLR4 activation.

IL6 and TNFA are associated with NOD-like receptor signaling pathway (Table 2). NOD-like receptors (NLRs) contribute significantly to the pathogenesis of obesity mediated insulin resistance by inducing proinflammatory cytokines^92^. Inflammatory cytokines induced by NLRs play role in diabetic retinopathy^93^ and progression to chronic kidney disease^94^.

As shown in Table 2, NOS3 and TNFA participate in sphingolipid signaling pathway. TNFA can stimulate sphingolipid (ceramide) generation^95^ and insulin resistance is promoted by elevated sphingolipid loads in the diabetic pancreas^96^. More research approaches are needed to investigate the concurrence of increased TNFA and sphingolipids in insulin resistance pathways.

Based on the IMEx predicted network (Fig. 1A) ZBTB16 connects between AGTR1 and IL6. ZBTB16, also known as promyelocytic leukemia zinc finger (PLZF), is a transcription factor (TF) that is be related to cardiac hypertrophy and/or fibrosis associated with hypertension and connected to components of metabolic syndrome such as dyslipidemia and insulin resistance via TNFA and IL6^97^. The level of serum alpha-2-macroglobulin (A2M), a protease inhibitor^98^, which is the connector between IL6, NOS3 and TNF in the network, is known to be elevated in diabetes^99^, retinopathy^100^ and nephrotic syndrome^101^. Further research focusing on whether there is any significant contribution of these two components (ZBTB16 and A2M) to diabetes and associated complications is needed. These connectors may help developing novel therapeutic strategies rather than targeting the individual nodes.

### Distribution patterns of variant allele and haplotype frequencies in populations of different ethnic orogins

The 1000 Genomes Project reconstructed the genomes of 2504 individuals from 26 populations from five super-populations (African (AFR), East Asian (EAS), European (EUR), South Asian (SAS), and admixed Americans (AMR)) with the aim of providing a comprehensive description of the human genetic variants^102^. Earlier studies revealed genetic stratifications among populations of different ethnicities and geographic distributions^103^. Global human population has gone through a number of stratifications and divisions during the course of evolution and migration. Within these subdivisions, people remixed and settled in widely varying environments, which resulted in the shuffling of genes leading to genetic heterogeneity. That is why allele frequency and haplotype analyses are important to understand population specific risk alleles.

Table 1 shows the different patterns in the distribution of variant alleles at rs1800629, rs1800795, rs1799983 and rs5186 loci in populations of different ethnicities. As mentioned earlier, the C allele at the rs5186 locus affects translation of AGTR1 leading to increased activation of the renin–angiotensin–aldosterone system. The range of MAFs for this variant is 0.207–0.266 in the admixed American populations and 0.207–0.313 in the European populations, whereas in other populations (Africans, East and South Asians) the range is 0.005 to 0.118. In the European populations, the variant allele frequencies of rs1799983 and rs1800795 are higher than the other populations. This might result in low circulating level of vasodilator NO by the variant allele itself or its linkage with the causal variant(s). For Utah residents with Northern and Western European ancestry and Finnish in Finland of European super-population, the MAFs at rs1800795 are close to 0.5. This variant allele was reported to be associated with insulin resistance, type 2 diabetes and myocardial injury^54,104^. The haplotype with two variant alleles (C_T) at rs1800795 and rs1799983 (two variants of chromosome 7) has significantly higher frequencies in European populations. Frequencies of this particular haplotype range from 0.101 to 0.182 in the European populations, whereas in other populations this range is between 0.002 and 0.087. At these loci the MAFs are >  0.2 in two admixed American sub-populations (Puerto Ricans from Puerto Rico and Colombians from Medellin, Colombia). Latin American populations are characterized by high level of genetic admixture among the Africans, Europeans and native American ancestral populations^105^. As shown in Table 1, among the four Latin American populations in Colombia, Mexico, Peru, and Puerto Rico- the pattern of variant allele frequencies in Puerto Ricans and Colombians is similar to the European populations, which might be because these two population inherited more genetic content from European ancestry than the Mexicans and the Peruvians^103^.

rs1800629, rs1800795, rs1799983 and rs5186 variants have low allele and haplotype frequencies in the African populations (Table 1). The pattern of MAFs at rs5186 in the two populations with African ancestry, but now residing in America [Americans of African Ancestry (ASW) and African Caribbeans in Barbados (ACB)] are similar to the African populations. The reason could be that the African Americans inherited approximately 80% of their genome from African ancestor and 20% from the Europeans^103^. The ancestors of African Americans and the African Caribbeans in Barbados were mostly from West Africa^103^. It was reported earlier that the East Asian and the African populations along with the Americans of African ancestry in USA (ASW) and the African Caribbeans in Barbados form completely different cluster from rest of the world populations^103^. The African populations, however, have relatively higher frequencies of other potentially deleterious variants in AGTR1, IL6, NOS3 and TNFA (Supplementary Table S1).

### Drug responses and distribution of variants in populations of different ethnicities

Approved drugs, that target these candidate genes, are listed in Table 3. Ethnic differences in allele frequencies at drug response-related SNP loci contribute to inter-population variability in drug response^103^. Three (rs5186, rs1800629 and rs1800795) of the four SNPs identified to be associated with diabetes and its complications can affect responses to drugs (Table 4). rs5186 is associated with the efficacies of losartan and angiotensin II (Table 4). Carriers of the variant allele (C) at rs5186 may have better humoral and renal hemodynamic responses to losartan treatment compared to individuals with the AA genotype^106^. High MAFs (≥ 0.2) at rs5186 in the admixed American and the European populations may make losartan a more effective drug for improving renal functions.

Individuals with the major allele G at rs1800629 show better response to anti-TNFA treatments in comparison to the carriers of the variant allele A^107^. Hence, individuals with increased TNFA level unsurprisingly show decreased response to anti-TNFA treatments. Efficacy of anti-TNFA treatment may also depend on the individual’s genotype at rs1800795- an SNP that affects IL6 expression level. Obese psoriasis patients with the major allele G at rs1800795 show decreased response to TNFA inhibitors^108^. Apart from the European populations and two admixed American populations (CLM and PUR), MAFs at rs1800795 in most populations, especially in the East Asians, is very low. Further studies are needed to fully elucidate how these differences in allele frequencies contribute to interpopulation differences in response to anti-TNFA therapeutics. Protein–protein interactions exist between TNFA and IL6 (Fig. 1). As previously discussed, obesity may influence plasma TNFA level. These complex interactions may explain why SNPs in IL6 may modulate response to TNFA inhibitors.

Since rs1799983 resides on the same chromosome with rs1800795 and rs1045642, all three were considered during haplotype frequency calculation (Supplementary Table S2). The haplotype with the major alleles at three SNP loci (G_G_G) is the most frequent in the African, admixed American and East Asian super-populations. The most common haplotype (G_A_G) in these the European and the South Asian super-populations contain the variant allele (A) at rs1045642. In these super-populations, the variant allele frequencies at this locus are ≥ 0.5. Hypertensive patients with rs1045642 A allele may have better response to losartan^109^. Again, efficacy of losartan might be more in treating diabetes associated complications in the European populations. Besides, rs1045642 is located in the ABCB1 (ATP‐binding cassette, subfamily B) gene, which encodes a drug efflux transporter^110^. Haplotype containing the variant alleles at all SNP loci (C_A_T) has a higher frequency (0.089) in the European super-population compared to the other populations. Thus, this haplotype calls for further investigation considering its association with diseases and drug-responses. Haplotype (C_G_T) with the variant alleles at rs1800795 (C) and rs1799983 (T) loci also has a higher frequency (0.062) in European super-population.

In addition to their contributions in diabetes and associated complications, TNFA, IL6, AGTR1 and NOS3 may play roles in other diseases^111^ (Supplementary Fig. S2). Drugs targeted to these genes might also have therapeutic benefits for other diseases with the same cause. For example, increased peripheral or central IL6 levels contributes to stress reaction and depressive disorder for which tocilizumab (antibody against IL6) mediated therapy can be a strategy to reduce the IL6 activity^112^.

In conclusion, the four genetic variants (rs5186, rs1800629, rs1799983, rs1800795) exert pleiotropic effects that can influence diabetes and associated complications and reside in genes that participate in pathways leading to these complications. These variants might also modulate other pathophysiological pathways. Based on the variant allele and haplotype frequencies, the European and admixed American populations have higher prevalence of the disease associated alleles at rs5186, rs1799983 and rs1800795 loci, which might make these populations more vulnerable to the diabetes and associated complications. These variants might modulate responses to approved drugs, and therefore, inter-population variability in response to drugs must be regarded. Early genetic characterization will give scope for more effective personalized treatment strategies. With prior knowledge and better understanding of risk factors and population specific haplotypes diagnostic tools and algorithms may be developed or improvised for different populations and preventive treatment strategies may be implicated to halt progression to severe complications.

## Materials and methods

### Identification of the common SNPs and associated genes

SNPCurator^113^ and SNP4Disease^114^ databases were searched with medical subject headings (MeSH)- “Diabetes mellitus”, “Cardiovascular diseases”, “Diabetic nephropathy”, “Diabetic retinopathy”, “Hypertension”, “Inflammation” and “Kidney diseases” to retrieve the list of SNPs associated with these conditions. SNPcurator is text mining system that extracts information from the genome wide and candidate genes studies^113^. The SNP4Disease database- developed by the Max Planck Institute for Heart and Lung Research, provides information on disease associated SNPs by literature-mining techniques from various sources^114^. With SNPCurator p < 0.05 was used to select SNPs. With SNP4Disease, it was not possible to filter based on p-values. There is wide variation in the sample sizes in different studies. It was not considered as a selection criterion during data retrieval. SNPs obtained for a particular MeSH term were merged and duplicates were removed. SNPs that are common to all MeSH terms were identified. Ultimately, four SNPs were identified to be commonly involved in the above mentioned conditions.

### Allele and haplotype frequencies of the common variants

LDhap tool at the open source LDlink suite^115^ was used for retrieving minor (variant) allele frequencies (MAF) of the SNPs and haplotype frequencies (of two SNPs that reside on the same chromosome) in five super-populations (African, admixed Americans, European East Asian, and South Asian) along with 26 constituent sub-populations. LDlink is a web-based collection of bioinformatic modules that query SNPs in population groups of interest for generating haplotype tables and calculating pair-wise linkage disequilibrium (LD) using the Phase 3 haplotype data from the 1000 Genomes Project as a reference^115^. Other SNPs within the candidate genes, that harbor the common variants, in the above mentioned 26 populations were retrieved using the Ensembl allele frequency calculator^116^.

### Determining phenotypic effects of the variants

PolyPhen-2^117^ and SIFT^118^ tools within the Ensembl variant effect predictor (VEP) were used to predict the effect of the genetic variants^119^. Only potentially deleterious/damaging variants were selected for further analysis. PolyPhen-2 predicts the functional significance of an allele replacement from its individual features by Naïve Bayes classifier trained using supervised machine-learning based on two pairs of datasets- HumDiv and HumVar^117^. In addition to calculating the probability whether a mutation is damaging by estimating the false positive rate (FPR, the chance that the mutation is classified as damaging when it is in fact non-damaging) and the true positive rate (TPR, the chance that the mutation is classified as damaging when it is indeed damaging), PolyPhen-2 also classifies a mutation qualitatively as- benign**, **possibly damaging**,** or probably damaging based on false positive rate (FPR) thresholds. On the other hand, SIFT is a multistep algorithm that predicts whether an amino acid substitution affects protein function based on sequence homology and the physical properties of amino acids^118^. SIFT scores ranges from 0 to 1. The amino acid substitution is predicted deleterious (affects protein function), if the score is < 0.05, and tolerated (neutral) if the score is ≥ 0.05. More details on the principle and procedure of calculating the PolyPhen-2 and SIFT scores are described in Adzhubei et al.^117^ and Vaser et al.^118^, respectively.

### Protein–protein network analysis

NetworkAnalyst^120^ web-interface was used to visualize the interactions among the gene products based on the protein–protein interaction data in the International Molecular Exchange Consortium (IMEx) database (using the default parameters and first-order network), which is a curated database containing non-redundant set of interaction data from a broad taxonomic range of organisms^29^. The modes of regulation among these gene products were retrieved using the STRING database using the default parameters^30^. The pathways that incorporate these gene products (with false discovery rate (FDR) ≤ 0.05) were retrieved from the KEGG pathway database^121^.

### Disease-gene interaction analysis

DisGeNET, one of the largest publicly accessible collections of genes and variants associated with human diseases^111^, was used via the NetworkAnalyst^120^ platform to visualize all possible associations of the candidate genes with human diseases.

### Analysis of drug-response

DGIdb^122^ and DrugBank^123^ databases were searched for the drugs that act on the candidate genes. Only the approved drugs were selected. List of SNPs associated with responses to the approved drugs targeting the candidate genes was retrieved from the pharmacogenomics knowledgebase^124^. Chromosomal positions and functional consequences of the SNPs were collected from dbSNP^125^. Alleles and minor allele frequencies of SNPs present in the 1000 Genomes Project were collected via Ensembl^116^. Haplotype frequencies were calculated with LDhap module of LDlink^115^. Only haplotypes with frequency of ≥ 0.05 in at least one super-population were listed.

### Statement of ethics

This study did not use human or animals. No ethical approval is required.

## Supplementary Information

Supplementary Information.
