# Supplementary material for: Common genetic variants and pathways in diabetes and associated complications and vulnerability of populations with different ethnic origins
Source: Sci Rep. 2021 Apr 5;11:7504. doi: 10.1038/s41598-021-86801-2 (PMC8021559; doi:10.1038/s41598-021-86801-2)
Supplement: Supplementary file 1 — Supplementary Information. [file 41598_2021_86801_MOESM1_ESM.docx]

**Article Title:** Common genetic variants and pathways in diabetes and associated complications and vulnerability of populations with different ethnic origins

Author names:

Sabrina Samad Shoily^1^,

Tamim Ahsan^2^,

Kaniz Fatema^1^,

Abu Ashfaqur Sajib^1,^*

Author affiliations:

^1^Department of Genetic Engineering & Biotechnology, University of Dhaka, Dhaka, Bangladesh.

^2^Bangabandhu Sheikh Mujibur Rahman Maritime University, Dhaka,Bangladesh.

*Corresponding author

Email: abu.sajib@du.ac.bd (AAS)


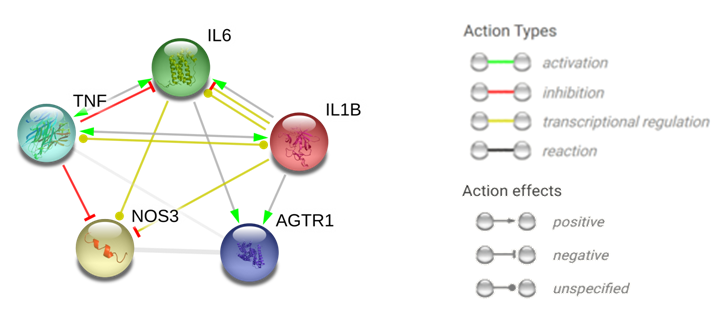


Supplementary Figure S1: Protein-protein interaction among interactions among AGTR1, IL1B IL6, NOS3 and TNFA.


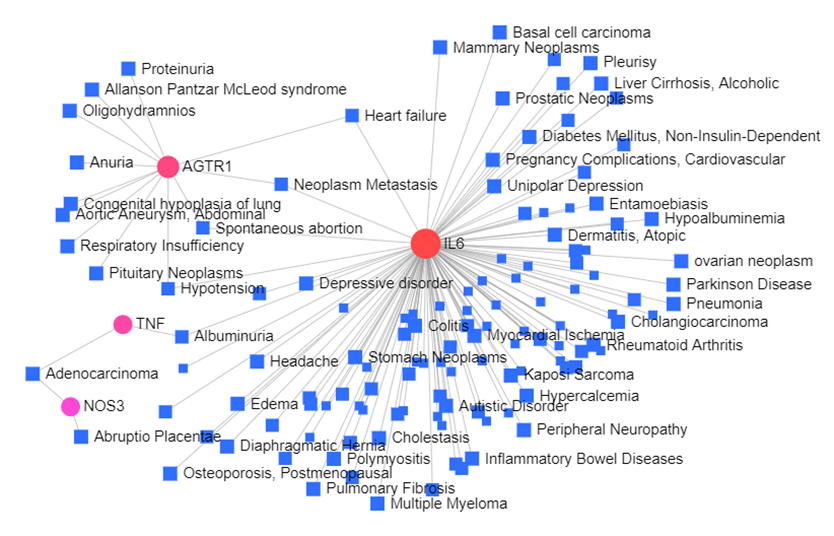


Supplementary Figure S2: Association of the candidate genes (IL6, TNFA, NOS3 and AGTR1) with other diseases.

Supplementary Table S1: Frequencies of the other potentially deleterious SNPs in AGTR1, IL-6, NOS3 and TNFA in different populations.

| Chromosome | Position | SNP ID | Gene | Reference allele | Variant allele | **Variant allele frequency** | | | | | | | | | | | | | | | | | | | | | | | | | |
| --- | --- | --- | --- | --- | --- | --- | --- | --- | --- | --- | --- | --- | --- | --- | --- | --- | --- | --- | --- | --- | --- | --- | --- | --- | --- | --- | --- | --- | --- | --- | --- |
|  |  |  |  |  |  | **AFR** | | | | | | | **AMR** | | | | **EAS** | | | | | **EUR** | | | | | **SAS** | | | | |
|  |  |  |  |  |  | YRI | LWK | GWD | MSL | ESN | ASW | ACB | MXL | PUR | CLM | PEL | CHB | JPT | CHS | CDX | KHV | CEU | TSI | FIN | GBR | IBS | GIH | PJL | BEB | STU | ITU |
| 3 | 148459015 | rs111980524 | AGTR1 | G | A | **0.01** | **0.02** | 0 | 0 | 0 | **0.02** | 0 | 0 | 0 | 0 | 0 | 0 | 0 | 0 | 0 | 0 | 0 | 0 | 0 | 0 | 0 | 0 | 0 | 0 | 0 | 0 |
| 3 | 148459090 | rs570903965 | AGTR1 | A | G | 0 | 0 | 0 | 0 | 0 | 0 | 0 | 0 | 0 | 0 | 0 | 0 | 0 | 0 | **0.01** | 0 | 0 | 0 | 0 | 0 | 0 | 0 | 0 | 0 | 0 | 0 |
| 3 | 148459166 | rs550259107 | AGTR1 | G | C | 0 | 0 | 0 | 0 | 0 | 0 | 0 | 0 | 0 | 0 | 0 | 0 | 0 | 0 | 0 | 0 | 0 | 0 | 0 | 0 | 0 | 0 | **0.01** | 0 | 0 | 0 |
| 3 | 148459241 | rs570056677 | AGTR1 | G | A | 0 | 0 | 0 | 0 | 0 | 0 | 0 | 0 | 0 | 0 | 0 | 0 | 0 | 0 | 0 | 0 | 0 | 0 | 0 | 0 | 0 | 0 | 0 | 0 | 0 | 0 |
| 3 | 148459322 | rs200184769 | AGTR1 | G | A | 0 | 0 | 0 | 0 | 0 | 0 | 0 | 0 | 0 | 0 | 0 | 0 | 0 | 0 | 0 | 0 | 0 | 0 | **0.01** | 0 | 0 | 0 | 0 | 0 | 0 | 0 |
| 3 | 148459349 | rs202211101 | AGTR1 | A | G | 0 | 0 | 0 | 0 | 0 | 0 | 0 | 0 | 0 | 0 | 0 | 0 | 0 | 0 | 0 | 0 | 0 | 0 | 0 | 0 | 0 | 0 | 0 | 0 | 0 | 0 |
| 3 | 148459384 | rs200844345 | AGTR1 | A | T | 0 | 0 | 0 | 0 | 0 | 0 | 0 | 0 | 0 | 0 | 0 | 0 | 0 | 0 | 0 | 0 | 0 | 0 | 0 | 0 | 0 | 0 | 0 | 0 | 0 | 0 |
| 3 | 148459552 | rs12721225 | AGTR1 | G | T | 0 | 0 | 0 | 0 | 0 | 0 | 0 | 0 | 0 | 0 | 0 | 0 | 0 | 0 | 0 | 0 | 0 | 0 | 0 | **0.01** | 0 | 0 | 0 | **0.01** | **0.03** | 0 |
| 3 | 148459558 | rs188281474 | AGTR1 | G | C | 0 | 0 | 0 | 0 | 0 | 0 | 0 | 0 | 0 | 0 | 0 | 0 | 0 | 0 | 0 | 0 | 0 | 0 | 0 | 0 | 0 | 0 | 0 | 0 | 0 | 0 |
| 3 | 148459748 | rs549928178 | AGTR1 | T | G | 0 | 0 | 0 | 0 | 0 | 0 | 0 | 0 | 0 | 0 | **0.01** | 0 | 0 | 0 | 0 | 0 | 0 | 0 | 0 | 0 | 0 | 0 | 0 | 0 | 0 | 0 |
| 3 | 148459776 | rs529403958 | AGTR1 | A | C | 0 | 0 | 0 | 0 | 0 | 0 | 0 | 0 | 0 | 0 | 0 | 0 | 0 | 0 | 0 | 0 | 0 | 0 | 0 | 0 | 0 | 0 | 0 | 0 | 0 | 0 |
| 3 | 148459840 | rs548345422 | AGTR1 | C | A | 0 | 0 | 0 | 0 | 0 | 0 | 0 | 0 | 0 | 0 | 0 | 0 | 0 | 0 | 0 | **0.01** | 0 | 0 | 0 | 0 | 0 | 0 | 0 | 0 | 0 | 0 |
| 6 | 31543541 | rs201502336 | TNFA | G | A | 0 | 0 | 0 | 0 | 0 | **0.01** | 0 | 0 | 0 | 0 | 0 | 0 | 0 | 0 | 0 | 0 | 0 | 0 | 0 | 0 | 0 | 0 | **0.01** | 0 | 0 | 0 |
| 6 | 31543574 | rs377338702 | TNFA | A | G | 0 | 0 | 0 | 0 | **0.01** | 0 | 0 | 0 | 0 | 0 | 0 | 0 | 0 | 0 | 0 | 0 | 0 | 0 | 0 | 0 | 0 | 0 | 0 | 0 | 0 | 0 |
| 6 | 31543604 | rs576621666 | TNFA | G | A | 0 | 0 | 0 | 0 | 0 | 0 | 0 | 0 | 0 | 0 | 0 | 0 | 0 | 0 | 0 | 0 | 0 | 0 | 0 | 0 | 0 | 0 | 0 | 0 | 0 | 0 |
| 6 | 31543639 | rs558678940 | TNFA | G | A | 0 | 0 | 0 | 0 | 0 | 0 | 0 | 0 | 0 | 0 | 0 | 0 | 0 | 0 | 0 | 0 | 0 | 0 | 0 | 0 | 0 | 0 | 0 | 0 | 0 | 0 |
| 6 | 31544571 | rs190788828 | TNFA | A | C | 0 | 0 | 0 | 0 | 0 | 0 | 0 | 0 | 0 | **0.01** | 0 | 0 | 0 | 0 | 0 | 0 | 0 | 0 | 0 | 0 | 0 | 0 | 0 | 0 | 0 | 0 |
| 6 | 31544961 | rs548532642 | TNFA | G | A | 0 | 0 | 0 | 0 | 0 | 0 | **0.01** | 0 | 0 | 0 | 0 | 0 | 0 | 0 | 0 | 0 | 0 | 0 | 0 | 0 | 0 | 0 | 0 | 0 | 0 | 0 |
| 6 | 31545093 | rs373646181 | TNFA | G | A | 0 | 0 | 0 | 0 | 0 | 0 | 0 | 0 | 0 | 0 | 0 | 0 | 0 | 0 | **0.01** | **0.02** | 0 | 0 | 0 | 0 | 0 | 0 | 0 | 0 | 0 | 0 |
| 6 | 31545189 | rs180710258 | TNFA | C | T | 0 | **0.01** | 0 | 0 | 0 | 0 | 0 | 0 | 0 | 0 | 0 | 0 | 0 | 0 | 0 | 0 | 0 | 0 | 0 | 0 | 0 | 0 | 0 | 0 | 0 | 0 |
| 7 | 150690910 | rs147771478 | NOS3 | G | A | 0 | 0 | 0 | 0 | 0 | 0 | 0 | 0 | 0 | 0 | 0 | 0 | 0 | 0 | 0 | 0 | 0 | 0 | 0 | 0 | 0 | 0 | 0 | 0 | 0 | 0 |
| 7 | 150690919 | rs547845670 | NOS3 | G | A | 0 | 0 | 0 | 0 | 0 | 0 | 0 | 0 | 0 | 0 | 0 | 0 | 0 | 0 | 0 | 0 | 0 | 0 | 0 | 0 | 0 | 0 | 0 | 0 | 0 | 0 |
| 7 | 150690970 | rs372106353 | NOS3 | G | A | 0 | 0 | 0 | 0 | 0 | 0 | 0 | 0 | 0 | 0 | 0 | 0 | 0 | 0 | 0 | 0 | 0 | 0 | 0 | 0 | 0 | 0 | 0 | 0 | 0 | 0 |
| 7 | 150690992 | rs556096751 | NOS3 | C | T | 0 | 0 | 0 | 0 | 0 | 0 | 0 | 0 | 0 | 0 | 0 | 0 | 0 | 0 | 0 | 0 | 0 | 0 | **0.01** | 0 | 0 | 0 | 0 | 0 | 0 | 0 |
| 7 | 150692293 | rs201405443 | NOS3 | C | G | 0 | 0 | 0 | 0 | 0 | 0 | 0 | 0 | 0 | 0 | 0 | 0 | 0 | 0 | 0 | 0 | 0 | 0 | 0 | 0 | 0 | 0 | 0 | 0 | 0 | 0 |
| 7 | 150693603 | rs143324164 | NOS3 | C | T | 0 | 0 | 0 | 0 | **0.01** | 0 | **0.01** | 0 | 0 | 0 | 0 | 0 | 0 | 0 | 0 | 0 | 0 | 0 | 0 | 0 | 0 | 0 | 0 | 0 | 0 | 0 |
| 7 | 150693869 | rs553156572 | NOS3 | C | T | 0 | 0 | 0 | 0 | 0 | 0 | 0 | 0 | 0 | 0 | 0 | 0 | 0 | 0 | 0 | 0 | 0 | 0 | 0 | 0 | 0 | 0 | 0 | 0 | 0 | 0 |
| 7 | 150693897 | rs141456642 | NOS3 | G | A | 0 | 0 | 0 | 0 | 0 | 0 | 0 | 0 | 0 | 0 | 0 | 0 | 0 | 0 | 0 | 0 | 0 | 0 | 0 | 0 | 0 | 0 | **0.01** | 0 | 0 | 0 |
| 7 | 150693947 | rs145711802 | NOS3 | C | A | 0 | 0 | 0 | 0 | 0 | 0 | 0 | 0 | 0 | 0 | 0 | 0 | 0 | 0 | 0 | 0 | 0 | 0 | 0 | 0 | 0 | 0 | 0 | 0 | 0 | 0 |
| 7 | 150696045 | rs142721517 | NOS3 | G | C | 0 | 0 | 0 | 0 | **0.01** | 0 | 0 | 0 | 0 | 0 | 0 | 0 | 0 | 0 | 0 | 0 | 0 | 0 | 0 | 0 | 0 | 0 | 0 | 0 | 0 | 0 |
| 7 | 150696049 | rs552501121 | NOS3 | G | A | 0 | 0 | 0 | 0 | **0.01** | 0 | 0 | 0 | 0 | 0 | 0 | 0 | 0 | 0 | 0 | 0 | 0 | 0 | 0 | 0 | 0 | 0 | 0 | 0 | 0 | 0 |
| 7 | 150696055 | rs562526133 | NOS3 | A | G | 0 | 0 | 0 | 0 | 0 | 0 | 0 | 0 | 0 | 0 | 0 | 0 | 0 | 0 | 0 | 0 | 0 | 0 | 0 | 0 | 0 | 0 | 0 | **0.01** | 0 | 0 |
| 7 | 150696076 | rs149539813 | NOS3 | G | A | 0 | 0 | 0 | 0 | 0 | 0 | 0 | 0 | 0 | 0 | 0 | 0 | 0 | 0 | 0 | 0 | 0 | 0 | **0.02** | 0 | 0 | 0 | 0 | 0 | 0 | 0 |
| 7 | 150696408 | rs572058640 | NOS3 | G | A | 0 | 0 | 0 | 0 | 0 | 0 | 0 | 0 | 0 | 0 | 0 | 0 | 0 | 0 | 0 | 0 | 0 | 0 | 0 | 0 | 0 | 0 | 0 | 0 | 0 | 0 |
| 7 | 150697652 | rs564627807 | NOS3 | G | A | 0 | 0 | 0 | 0 | 0 | 0 | 0 | 0 | 0 | 0 | 0 | 0 | 0 | 0 | 0 | 0 | 0 | 0 | 0 | 0 | 0 | 0 | 0 | 0 | 0 | **0.01** |
| 7 | 150698340 | rs555751737 | NOS3 | G | A | 0 | 0 | 0 | 0 | 0 | 0 | 0 | 0 | 0 | 0 | 0 | 0 | 0 | 0 | 0 | 0 | 0 | 0 | 0 | 0 | 0 | 0 | 0 | 0 | 0 | 0 |
| 7 | 150698352 | rs141089940 | NOS3 | G | A | 0 | 0 | 0 | 0 | 0 | 0 | 0 | 0 | 0 | 0 | 0 | 0 | 0 | 0 | 0 | 0 | 0 | 0 | 0 | 0 | 0 | 0 | 0 | 0 | 0 | 0 |
| 7 | 150698356 | rs578101703 | NOS3 | C | T | 0 | 0 | 0 | 0 | **0.01** | 0 | 0 | 0 | 0 | 0 | 0 | 0 | 0 | 0 | 0 | 0 | 0 | 0 | 0 | 0 | 0 | 0 | 0 | 0 | 0 | 0 |
| 7 | 150698385 | rs563793183 | NOS3 | G | A | 0 | 0 | 0 | 0 | **0.01** | 0 | 0 | 0 | 0 | 0 | 0 | 0 | 0 | 0 | 0 | 0 | 0 | 0 | 0 | 0 | 0 | 0 | 0 | 0 | 0 | 0 |
| 7 | 150698401 | rs375829467 | NOS3 | G | C | 0 | 0 | 0 | 0 | 0 | 0 | 0 | 0 | 0 | 0 | 0 | 0 | 0 | 0 | 0 | 0 | 0 | 0 | 0 | 0 | 0 | 0 | 0 | 0 | 0 | 0 |
| 7 | 150698923 | rs201216025 | NOS3 | C | T | 0 | 0 | 0 | 0 | 0 | 0 | 0 | 0 | 0 | 0 | 0 | 0 | 0 | 0 | 0 | 0 | 0 | 0 | 0 | 0 | 0 | 0 | 0 | 0 | 0 | 0 |
| 7 | 150699030 | rs139628271 | NOS3 | T | C | 0 | 0 | 0 | 0 | 0 | 0 | 0 | 0 | 0 | 0 | 0 | 0 | 0 | 0 | 0 | 0 | 0 | 0 | 0 | 0 | 0 | 0 | 0 | **0.01** | 0 | 0 |
| 7 | 150699316 | rs181342641 | NOS3 | T | C | 0 | 0 | 0 | 0 | 0 | 0 | 0 | 0 | 0 | 0 | 0 | 0 | 0 | 0 | 0 | 0 | 0 | 0 | 0 | 0 | 0 | 0 | 0 | 0 | 0 | 0 |
| 7 | 150700332 | rs528654243 | NOS3 | A | C | 0 | 0 | 0 | 0 | 0 | 0 | 0 | 0 | 0 | 0 | 0 | 0 | 0 | 0 | 0 | 0 | 0 | 0 | 0 | 0 | 0 | 0 | 0 | 0 | 0 | 0 |
| 7 | 150700776 | rs532240232 | NOS3 | C | T | 0 | 0 | 0 | 0 | 0 | 0 | 0 | 0 | 0 | 0 | 0 | 0 | 0 | **0.01** | 0 | 0 | 0 | 0 | 0 | 0 | 0 | 0 | 0 | 0 | 0 | 0 |
| 7 | 150704038 | rs570681394 | NOS3 | C | T | 0 | 0 | 0 | 0 | 0 | 0 | 0 | 0 | 0 | 0 | 0 | 0 | 0 | 0 | 0 | 0 | 0 | 0 | 0 | 0 | 0 | 0 | 0 | **0.01** | **0.01** | **0.01** |
| 7 | 150704083 | rs556539002 | NOS3 | G | A | 0 | 0 | 0 | 0 | 0 | 0 | 0 | 0 | 0 | 0 | 0 | 0 | 0 | 0 | 0 | 0 | 0 | 0 | 0 | 0 | 0 | 0 | 0 | 0 | 0 | 0 |
| 7 | 150704359 | rs563391462 | NOS3 | T | C | 0 | 0 | 0 | 0 | 0 | 0 | 0 | 0 | 0 | 0 | 0 | 0 | 0 | 0 | 0 | 0 | 0 | 0 | 0 | 0 | 0 | 0 | 0 | 0 | 0 | 0 |
| 7 | 150706075 | rs200106471 | NOS3 | T | C | 0 | 0 | 0 | 0 | 0 | 0 | 0 | 0 | 0 | 0 | 0 | 0 | 0 | 0 | 0 | 0 | 0 | 0 | 0 | **0.01** | 0 | 0 | 0 | 0 | 0 | 0 |
| 7 | 150706112 | rs544887797 | NOS3 | G | A | 0 | 0 | 0 | 0 | 0 | 0 | 0 | 0 | 0 | 0 | 0 | 0 | 0 | 0 | 0 | **0.01** | 0 | 0 | 0 | 0 | 0 | 0 | 0 | 0 | 0 | 0 |
| 7 | 150706321 | rs138716215 | NOS3 | C | T | 0 | 0 | 0 | 0 | 0 | 0 | 0 | 0 | 0 | 0 | 0 | 0 | 0 | 0 | 0 | 0 | 0 | 0 | 0 | 0 | 0 | **0.01** | 0 | 0 | 0 | 0 |
| 7 | 150706502 | rs200183107 | NOS3 | G | C | 0 | 0 | 0 | 0 | 0 | 0 | 0 | 0 | 0 | 0 | 0 | 0 | 0 | 0 | 0 | 0 | 0 | 0 | 0 | 0 | 0 | 0 | 0 | 0 | 0 | 0 |
| 7 | 150706545 | rs368180942 | NOS3 | C | T | 0 | 0 | 0 | 0 | 0 | 0 | 0 | 0 | 0 | 0 | 0 | 0 | 0 | 0 | **0.01** | **0.01** | 0 | 0 | 0 | 0 | 0 | 0 | 0 | **0.01** | 0 | 0 |
| 7 | 150706640 | rs3918232 | NOS3 | G | A | 0 | 0 | 0 | 0 | 0 | 0 | 0 | 0 | 0 | **0.01** | 0 | 0 | 0 | 0 | 0 | 0 | **0.01** | 0 | 0 | 0 | 0 | 0 | **0.01** | 0 | 0 | 0 |
| 7 | 150707251 | rs148919189 | NOS3 | C | T | 0 | 0 | **0.02** | 0 | 0 | 0 | 0 | 0 | 0 | 0 | 0 | 0 | 0 | 0 | 0 | 0 | 0 | 0 | 0 | 0 | 0 | 0 | 0 | 0 | 0 | 0 |
| 7 | 150707253 | rs145765422 | NOS3 | C | G | 0 | 0 | 0 | 0 | 0 | 0 | 0 | 0 | 0 | 0 | 0 | 0 | 0 | 0 | 0 | 0 | 0 | 0 | 0 | 0 | 0 | 0 | 0 | 0 | 0 | 0 |
| 7 | 150707314 | rs201601172 | NOS3 | G | A | 0 | 0 | 0 | 0 | 0 | 0 | 0 | 0 | 0 | 0 | 0 | 0 | 0 | 0 | 0 | 0 | 0 | 0 | 0 | 0 | 0 | 0 | 0 | 0 | 0 | 0 |
| 7 | 150707332 | rs200507709 | NOS3 | C | T | 0 | 0 | 0 | 0 | 0 | 0 | 0 | 0 | 0 | 0 | 0 | 0 | 0 | 0 | 0 | 0 | **0.01** | 0 | 0 | 0 | 0 | 0 | 0 | 0 | 0 | 0 |
| 7 | 150707344 | rs3918201 | NOS3 | G | T | **0.06** | **0.08** | **0.04** | **0.03** | **0.07** | **0.01** | **0.04** | 0 | 0 | 0 | **0.01** | 0 | 0 | 0 | 0 | 0 | 0 | 0 | 0 | 0 | 0 | 0 | 0 | 0 | 0 | 0 |
| 7 | 150707694 | rs182213442 | NOS3 | C | T | 0 | 0 | 0 | 0 | 0 | 0 | 0 | 0 | 0 | 0 | 0 | 0 | 0 | 0 | 0 | 0 | 0 | 0 | 0 | 0 | 0 | 0 | 0 | 0 | 0 | 0 |
| 7 | 150708046 | rs563005534 | NOS3 | G | A | 0 | 0 | 0 | 0 | 0 | 0 | 0 | 0 | 0 | 0 | 0 | 0 | 0 | 0 | 0 | **0.01** | 0 | 0 | 0 | 0 | 0 | 0 | 0 | 0 | 0 | 0 |
| 7 | 150708908 | rs540435018 | NOS3 | G | A,C | 0.0, 0.0 | 0.01, 0.0 | 0.0,0.0 | 0.0,  0.0 | 0.0,  0.0 | 0.0,  0.0 | 0.0,  0.0 | 0.0,0.01 | 0.0,  0.0 | 0.0,  0.0 | 0.0,  0.0 | 0.0,  0.0 | 0.0,  0.0 | 0.0,  0.0 | 0.0,  0.0 | 0.0,  0.0 | 0.0,  0.0 | 0.0,  0.0 | 0.0,  0.0 | 0.0, 0.01 | 0.0,  0.0 | 0.0,  0.0 | 0.0,  0.0 | 0.0,  0.0 | 0.0,  0.0 | 0.0,  0.0 |
| 7 | 150709468 | rs533251778 | NOS3 | C | A | 0 | 0 | 0 | 0 | 0 | 0 | 0 | 0 | 0 | 0 | 0 | 0 | 0 | 0 | **0.01** | 0 | 0 | 0 | 0 | 0 | 0 | 0 | 0 | 0 | 0 | 0 |
| 7 | 150710392 | rs3730011 | NOS3 | G | A | **0.03** | **0.03** | **0.02** | 0 | **0.02** | 0 | 0 | 0 | 0 | 0 | 0 | 0 | 0 | 0 | 0 | 0 | 0 | 0 | 0 | 0 | 0 | 0 | 0 | 0 | 0 | 0 |
| 7 | 22766886 | rs200700194 | IL-6 | A | T | 0 | 0 | 0 | 0 | 0 | 0 | 0 | 0 | 0 | 0 | 0 | 0 | 0 | 0 | 0 | 0 | 0 | 0 | 0 | 0 | 0 | 0 | 0 | 0 | 0 | 0 |
| 7 | 22766897 | rs569143621 | IL-6 | A | G | 0 | 0 | 0 | 0 | 0 | 0 | 0 | 0 | 0 | 0 | 0 | 0 | 0 | 0 | 0 | **0.01** | 0 | 0 | 0 | 0 | 0 | 0 | 0 | 0 | 0 | 0 |
| 7 | 22767009 | rs553784698 | IL-6 | A | T | 0 | 0 | 0 | 0 | 0 | 0 | **0.01** | 0 | 0 | 0 | 0 | 0 | 0 | 0 | 0 | 0 | 0 | 0 | 0 | 0 | 0 | 0 | 0 | 0 | 0 | 0 |
| 7 | 22767020 | rs376047818 | IL-6 | T | A | 0 | 0 | 0 | **0.01** | 0 | 0 | 0 | 0 | 0 | 0 | 0 | 0 | 0 | 0 | 0 | 0 | 0 | 0 | 0 | 0 | 0 | 0 | 0 | 0 | 0 | 0 |
| 7 | 22767056 | rs201493844 | IL-6 | G | C | 0 | 0 | 0 | 0 | 0 | **0.01** | 0 | 0 | 0 | 0 | 0 | 0 | 0 | 0 | 0 | 0 | 0 | 0 | 0 | 0 | 0 | 0 | 0 | 0 | 0 | 0 |
| 7 | 22767064 | rs199941251 | IL-6 | C | A | 0 | 0 | 0 | 0 | 0 | 0 | 0 | 0 | 0 | 0 | 0 | 0 | 0 | 0 | 0 | 0 | 0 | 0 | 0 | 0 | 0 | 0 | 0 | 0 | 0 | 0 |
| 7 | 22767134 | rs142759801 | IL-6 | C | A | **0.01** | **0.01** | **0.04** | **0.01** | **0.03** | **0.02** | **0.03** | 0 | 0 | 0 | 0 | 0 | 0 | 0 | 0 | 0 | 0 | 0 | 0 | 0 | 0 | 0 | 0 | 0 | 0 | 0 |
| 7 | 22768336 | rs190436077 | IL-6 | G | C | 0 | 0 | 0 | 0 | 0 | 0 | 0 | 0 | 0 | 0 | 0 | 0 | 0 | 0 | 0 | 0 | 0 | 0 | 0 | 0 | 0 | 0 | 0 | 0 | 0 | 0 |
| 7 | 22768412 | rs202210223 | IL-6 | C | A | 0 | 0 | 0 | 0 | 0 | 0 | 0 | 0 | 0 | 0 | 0 | 0 | 0 | 0 | 0 | 0 | 0 | 0 | 0 | 0 | 0 | 0 | 0 | 0 | 0 | 0 |
| 7 | 22769154 | rs148171375 | IL-6 | A | T | 0 | 0 | 0 | 0 | 0 | 0 | 0 | 0 | 0 | 0 | 0 | 0 | 0 | 0 | 0 | 0 | 0 | 0 | 0 | 0 | 0 | 0 | 0 | 0 | 0 | 0 |
| 7 | 22769264 | rs182812860 | IL-6 | G | C | 0 | 0 | 0 | 0 | 0 | 0 | 0 | 0 | 0 | **0.01** | 0 | 0 | 0 | 0 | 0 | 0 | 0 | 0 | 0 | 0 | 0 | 0 | 0 | 0 | 0 | 0 |
| 7 | 22769310 | rs2069842 | IL-6 | G | A | **0.13** | **0.07** | **0.1** | **0.1** | **0.07** | **0.05** | **0.09** | 0 | 0 | **0.01** | 0 | 0 | 0 | 0 | 0 | 0 | 0 | 0 | 0 | 0 | 0 | 0 | 0 | 0 | 0 | 0 |
| 7 | 22769364 | rs143808144 | IL-6 | C | T | 0 | 0 | 0 | 0 | 0 | 0 | 0 | 0 | 0 | 0 | 0 | 0 | 0 | 0 | 0 | 0 | **0.01** | 0 | 0 | 0 | 0 | 0 | 0 | 0 | 0 | 0 |
| 7 | 22769365 | rs539937012 | IL-6 | A | C | 0 | 0 | 0 | 0 | 0 | 0 | 0 | 0 | 0 | 0 | 0 | 0 | 0 | 0 | 0 | 0 | 0 | 0 | 0 | 0 | 0 | 0 | 0 | 0 | 0 | 0 |
| 7 | 22771156 | rs2069849 | IL-6 | C | T | **0.19** | **0.11** | **0.14** | **0.21** | **0.18** | **0.16** | **0.14** | **0.08** | **0.07** | **0.09** | **0.09** | 0 | 0 | 0 | **0.01** | 0 | **0.02** | **0.01** | **0.02** | **0.02** | **0.04** | **0.08** | **0.06** | **0.03** | **0.06** | **0.07** |

# African super-population (**AFR**)- ((Yoruba in Ibadan, Nigeria (**YRI**); Luhya in Webuye, Kenya (**LWK**); Gambian in Western Divisions in the Gambia (**GWD**); Mende in Sierra Leone (**MSL**); Esan in Nigeria (**ESN**); Americans of African Ancestry in SW USA (**ASW**); African Caribbeans in Barbados (**ACB**)); Admixed Americans (**AMR**)- ((Mexican Ancestry from Los Angeles USA (**MXL**); Puerto Ricans from Puerto Rico (**PUR**); Colombians from Medellin, Colombia (**CLM**); Peruvians from Lima, Peru (**PEL**)); East Asian (**EAS**)- ((Han Chinese in Beijing, China (**CHB**); Japanese in Tokyo, Japan (**JPT**); Southern Han Chinese (**CHS**); Chinese Dai in Xishuangbanna, China (**CDX**); Kinh in Ho Chi Minh City, Vietnam (**KHV**)); European (**EUR**)- ((Utah Residents (**CEPH**) with Northern and Western European Ancestry (**CEU**); Toscani in Italia (**TSI**); Finnish in Finland (FIN); British in England and Scotland (**GBR**); Iberian Population in Spain (**IBS**)); South Asian (**SAS**)- ((Gujarati Indian from Houston, Texas (**GIH**); Punjabi from Lahore, Pakistan (**PJL**); Bengali from Bangladesh (**BEB**); Sri Lankan Tamil from the UK (**STU**); Indian Telugu from the UK (**ITU**))

Supplementary Table S2: Haplotype frequencies of drug response-related SNPs on chromosome 7.

| Chromosome | SNP ID | Drugs | Haplotypes^a^ | Frequency^b^ | | | | | |
| --- | --- | --- | --- | --- | --- | --- | --- | --- | --- |
|  |  |  |  | ALL | AFR | AMR | EAS | EUR | SAS |
| 7 | rs1800795_rs1045642_rs1799983 | Losartan, adalimumab, infliximab, etanercept | G_G_G | 0.463 | 0.781 | 0.382 | 0.523 | 0.195 | 0.303 |
|  |  |  | G_***A***_G | 0.262 | 0.132 | 0.272 | 0.346 | 0.196 | 0.411 |
|  |  |  | G_G_***T*** | 0.075 | 0.053 | 0.087 | 0.079 | 0.102 | 0.061 |
|  |  |  | G_***A_T*** | ***0.06*** | 0.014 | ***0.075*** | ***0.051*** | ***0.092*** | ***0.086*** |
|  |  |  | ***C_A***_G | ***0.051*** | 0.002 | ***0.062*** | 0.001 | ***0.142*** | ***0.068*** |
|  |  |  | ***C***_G_G | 0.048 | 0.014 | 0.069 |  | 0.123 | 0.051 |
|  |  |  | ***C_A_T*** | 0.023 | 0.001 | 0.019 |  | ***0.089*** | 0.01 |
|  |  |  | ***C***_G_***T*** | 0.02 | 0.002 | 0.034 |  | ***0.062*** | 0.01 |

^a^Variant alleles are written in bold italic fonts.

^b^Haplotype with ≥2 variant alleles and frequencies ≥ 0.05 are written in bold italic letters.
